# Cisplatin and doxorubicin chemotherapy alters gut microbiota in a murine osteosarcoma model

**DOI:** 10.18632/aging.205428

**Published:** 2024-01-16

**Authors:** Zhi Tian, Xiaochen Qiao, Zhichao Wang, Xiaoyan Li, Yongchun Pan, Xiaochun Wei, Zhi Lv, Pengcui Li, Qiujing Du, Wenhao Wei, Lei Yan, Song Chen, Chaojian Xu, Yi Feng, Ruhao Zhou

**Affiliations:** 1 Second Clinical Medical College, Shanxi Medical University, Taiyuan, Shanxi 030001, P.R. China; 2 Department of Orthopedics, The Second Hospital of Shanxi Medical University, Shanxi Key Laboratory of Bone and Soft Tissue Injury Repair, Taiyuan, Shanxi 030001, P.R. China; 3 Third Hospital of Shanxi Medical University, Shanxi Bethune Hospital, Shanxi Academy of Medical Sciences, Tongji Shanxi Hospital, Taiyuan 030032, P.R. China; 4 Department of Orthopedics, The Third People’s Hospital of Datong City, Datong, Shanxi 037006, P.R. China; 5 Department of Orthopedics, JinZhong Hospital Affiliated to Shanxi Medical University, Jinzhong, Shanxi 030600, P.R. China; 6 Shanxi Province Cancer Hospital, Shanxi Hospital Affiliated to Cancer Hospital, Chinese Academy of Medical Sciences, Cancer Hospital Affiliated to Shanxi Medical University, Taiyuan, Shanxi 030013, P.R. China

**Keywords:** osteosarcoma, gut microbiota, chemotherapy, animal model, 16S rRNA sequencing

## Abstract

The gut microbiota is closely associated with tumor progression and treatment in a variety of cancers. However, the alteration of the gut microbiota during the progression and chemotherapy of osteosarcoma remains poorly understood. This study aimed to explore the relationship between dysbiosis in the gut microbiota during osteosarcoma growth and chemotherapy treatment. We used BALB/c nude mice to establish osteosarcoma xenograft tumor models and administered cisplatin (CDDP) or doxorubicin (DOX) intraperitonially once every 2 days for a total of 5 times to establish effective chemotherapy models. Fecal samples were collected and processed for 16S rRNA sequencing to analyze the composition of the gut microbiota. We observed that the abundances of *Colidextribacter*, *Lachnospiraceae_NK4A136*_group, *Lachnospiraceae_UCG-010*, *Lachnospiraceae_UCG-006*, and *Lachnoclostridium* decreased, and the abundances of *Alloprevotella* and *Enterorhabdus* increased in the osteosarcoma mouse model group compared to those in the control group. In addition, genera, such as *Lachnoclostridium* and *Faecalibacterium* were more abundant in chemotherapy-treated mice than those in saline-treated mice. Additionally, we observed that alterations in some genera, including *Lachnoclostridium* and *Colidextribacter* in the osteosarcoma animal model group returned to normal after CDDP or DOX treatment. Furthermore, the function of the gut microbiota was inferred through PICRUSt2 (Phylogenetic Investigation of Communities by Reconstruction of Unobserved States), which indicated that metabolism-related microbiota was highly enriched and significantly different in each group. These results indicate correlations between dysbiosis of the gut microbiota and osteosarcoma growth and chemotherapy treatment with CDDP or DOX and may provide novel avenues for the development of potential adjuvant therapies.

## INTRODUCTION

Osteosarcoma is the most common primary malignant solid tumor of the bone, accounting for approximately 20% of the primary malignant bone tumors. Osteosarcoma, common among adolescents aged 10–25 years, is known to have a high degree of malignancy and is prone to early lung metastasis [1]. Despite advances in chemotherapy and postoperative adjuvant therapy, the 5-year survival rate for metastatic and recurrent osteosarcoma is only 20% [2, 3].

Gut microbiota plays an important role in human health and disease. Cho and Costello suggest that the human microbiome consists of a variety of microorganisms, such as bacteria, fungi, archaea, protozoa, and viruses that inhabit the surface of the human epithelial barrier [4, 5]. Gut microbiota refers to the microbiota in the intestinal tract and consists of a variety of organisms with complex classifications. Gut microbiota is associated with host metabolism and immunity [6]. Recently, studies have found that the microbiome is closely associated with the occurrence and development of various cancers; for example, *Helicobacter pylori* is recognized by the World Health Organization as a type I carcinogen and is the strongest single risk factor for gastric cancer [7]. Additionally, the abundance of *Faecalibacterium prausnitzii* and *Blautia sp.* was correlated with the degree of breast cancer malignancy [8]. Gut microbiota has been shown to support cancer stem cell survival, proliferation, and promote intestinal tumor development in mice by activating the calcineurin- nuclear factor of activated T-cells pathway [9]. Butyrate-producing bacteria in the gut ferment dietary fiber to produce butyrate, which suppresses colonic inflammation and carcinogenesis by activating Gpr109a [10]. However, the specific microbes associated with the occurrence and development of osteosarcoma remain unknown.

Recently, the importance of gut microbiota in cancer treatment has become increasingly important [11–13]. Cyclophosphamide, a clinically important chemotherapeutic drugs, alters the composition of gut microbiota in mice, and these alterations help shape the anticancer immune response [14, 15]. Cisplatin (CDDP) and doxorubicin (DOX) are DNA-damaging cytotoxic agents that are first-line chemotherapeutic agents used to treat osteosarcoma [16]. Adjuvant chemotherapy following surgical resection of localized osteosarcoma has significantly improved the 5-year survival rate to 60–70 percent [17]. We hypothesized that microbiota dysbiosis induced by chemotherapeutic agents may be one of the mechanisms underlying the antitumor effect of chemotherapy. However, CDDP- or DOX-induced alterations in the gut microbiota during osteosarcoma treatment have not been investigated.

Consequently, we hypothesized that alterations in the gut microbiota are associated with osteosarcoma progression and chemotherapy. To verify this hypothesis, we evaluated changes in gut microbiota during osteosarcoma growth and after chemotherapy treatment with CDDP or DOX in mouse models by analyzing fecal 16S rRNA and examined the associations between progression and chemotherapy of osteosarcoma and gut microbiota in mice. Our results provide new insights into the progression and treatment of osteosarcoma.

## MATERIALS AND METHODS

### Research animals and Saos-2 cell line

Four-week-old female BALB/c nude mice (*n* = 24) were purchased from Charles River Laboratory (Beijing, China). A well-controlled environment (24°C, 12 h light/dark cycles) was provided to all mice, and the animals had free access to food and drinking water. After adaptive feeding for one week, 24 mice were divided into four groups. The human osteosarcoma cell line, Saos-2 was obtained from the American Type Culture Collection (ATCC) and cultured in Dulbecco’s modified Eagle’s medium (DMEM) supplemented with 10% fetal bovine serum (FBS) in a humidified incubator with 5% CO_2_ at 37°C.

### Saos-2 transplantation and CDDP/DOX treatment

After acclimatization for one week, each mouse was randomly assigned to one of the four groups: control group (group A), osteosarcoma model group (group B), CDDP-treated group (group C), and DOX-treated group (group D); *n* = 6 mice per group. In groups B, C, and D, mice were subcutaneously implanted with 1 × 10^6^ Saos-2 cells mixed with 200 μL Matrigel (Corning, NY, USA) into the back flank, as previously described [18, 19]. Ten days after implantation, CDDP (Cat# HY-17394, MedChemExpress, Monmouth Junction, NJ, USA) and DOX (Cat# HY-15142, MedChemExpress, Monmouth Junction, NJ, USA) were dissolved in normal saline solution at a final concentration of 0.3 mg/mL. The CDDP-treated group C and DOX-treated group D were administered CDDP (3 mg/kg) or DOX (3 mg/kg) intraperitoneally once every 2 days for a total of 5 times, and groups A and B were administered equal volumes of normal saline (vehicle).

### Sample collection

Forty-eight hours after the last drug treatment, fresh feces from each mouse were collected in sterile freezing tubes within 6 h. The feces samples were snap-frozen in liquid nitrogen before being transferred to a refrigerator at −80°C for storage. All mice were humanely euthanized and the tumors were completely dissected and weighed.

### Immunohistochemistry

The osteosarcoma tumor samples from mice were fixed with 10% formalin for 1 day, paraffin-embedded and sectioned at 4 μm thickness. The sections were dewaxed, rehydrated, blocked with 5% bovine serum albumin (BSA), and incubated with an anti-Ki67 antibody (A2094, Abclonal, Wuhan, China) at 4°C for 16 h. The sections were then washed three times with PBS, incubated with horseradish peroxidase-conjugated goat anti-mouse IgG (ZSGB-BIO, Beijing, China) at 37°C for 1 h, and then counterstained with hematoxylin solution. The procedure described above has been described in a previous study [20]. The intensity of anti-Ki67 staining was analyzed using a scanner control software (Pannoramic MIDI II, 3DHISTECH, Budapest, Hungary).

### DNA extraction and amplification

Following the manufacturer’s instructions, bacterial DNA was extracted from fecal samples using the MagPure Soil DNA LQ Kit (Magen, Guangdong, China). The DNA concentration was measured using a NanoDrop 2000 spectrophotometer (Thermo Fisher Scientific, Waltham, MA, USA) and the DNA integrity was assessed using the agarose gel electrophoresis. Briefly, in a 25 μL reaction volume, universal primer pairs (343F:5′ TACGGRAGGCAGCAG 3′, 798R:5′ AGGGTATCTAATCCT 3′) were used to perform PCR amplification of the V3-V4 hypervariable regions of bacterial 16S rRNA genes. The reverse primer included a sample barcode and both primers were connected to an Illumina sequencing adapter.

### Library construction and 16S rRNA sequencing

16S rRNA sequencing was performed as previously described [21]. Gel electrophoresis was used to visualize the quality of the amplicon. The PCR products were purified using Agencourt AMPure XP beads (Beckman Coulter Co., USA) and quantified using a Qubit dsDNA assay kit. The concentrations of the PCR products were adjusted for sequencing. Sequencing was performed using an Illumina NovaSeq6000 with two paired-end read cycles of 250 bases each (Illumina Inc., San Diego, CA; OE Biotech Company, Shanghai, China).

### Bioinformatic analysis

Raw sequencing data were obtained in the FASTQ format. Paired-end reads were preprocessed using a Trimmomatic software [22] to detect and filter ambiguous bases (N). We used the sliding window trimming approach to filter low-quality sequences that scored below 20 on average. After trimming, the FLASH software [23] was used to assemble the paired-end reads. Briefly, 10 bp of minimal overlap, 200 bp of maximum overlap, and 20% of the maximum mismatch rate were regarded as the assembly parameters. We performed further denoising on the sequence as follows: reads with ambiguous, homologous sequences, or less than 200 bp were discarded, and reads with a minimum of 75% bases above a quality score (Q) of 20 were preserved. The QIIME software [24] (version 1.8.0) was used to detect and remove chimeric reads. Based on similarity cutoffs of 97%, operational taxonomic units (OTUs) were generated using the Vsearch software [25] after primer sequence removal and clustering of clean reads. We used the QIIME package to select representative OTU reads. The Silva database Version 138, RDP classifier, [26] was used to annotate all representative reads with a 70% confidence threshold. Alpha diversity, including the Shannon and Simpson indices, was employed to estimate the microbial diversity in the fecal samples. Euclidean principal coordinates analysis (PCoA) was performed based on the Euclidean distance matrix using the QIIME software.

### Statistical analysis

Statistical analyses were performed using the IBM SPSS Statistics software (version 21.0). Data from the groups are expressed as mean ± standard error of the mean (SEM). The comparison of the means of two independent samples was analyzed using two-tailed Student’s *t*-test, and one-way ANOVA was used to compare inter-group of samples. Differences were considered statistically significant at *P* < 0.05.

### Availability of data and materials

The raw sequence data generated in this study are available in the NCBI Sequence Read Archive (accession number PRJNA815947).

## RESULTS

We conducted a study to compare the differences between the gut microbiota of mice model with osteosarcoma and mice in the control groups, subjected to chemotherapy agents and saline, using 16S rRNA gene sequencing. It was found that the diversity and composition of the microbiota differed significantly between the groups. Furthermore, the gut microbiota may affect the progression of osteosarcoma and response to chemotherapy in the mice with osteosarcoma model through metabolic pathways.

### Effects of CDDP and DOX on tumor inhibition in mice

**Figure 1:**
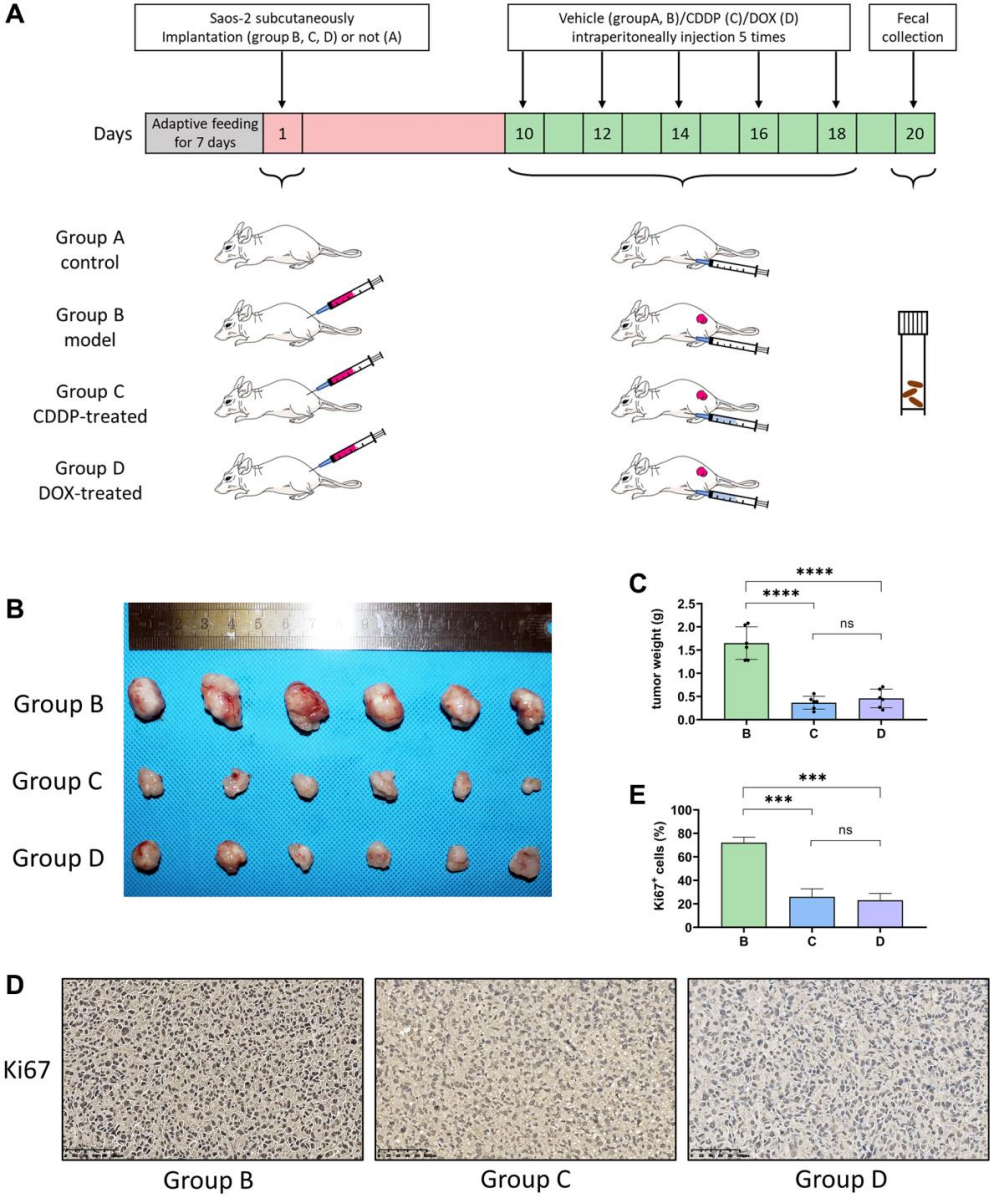
**CDDP and DOX inhibit the osteosarcoma tumor growth in mice.** (**A**) Outline of the experimental procedure. (**B**) Photographs of osteosarcoma tumor xenografts. Saos-2 cells were injected into nude mice, which were then treated with normal saline, CDDP, or DOX. After 20 days, osteosarcoma xenograft tumors were completely dissected and photographed. (**C**) Weight of the tumors. (^****^P < 0.0001; ns *P* > 0.05; *n* = 6). (**D**) Immunohistochemistry: Immunohistochemistry analyses were performed on mouse osteosarcoma xenograft tumor tissues. (**E**) The percentage of Ki67+ cells in the tumors. (^***^P < 0.001; ns *P* > 0.05; *n* = 6) Scale bar, 100 μm. Abbreviations: CDDP: cisplatin; DOX: doxorubicin.

The experimental procedure used in this study is illustrated in Figure 1A. The tumor volume in the CDDP-treated group (C) and DOX-treated group (D) was significantly lesser than that in the model group (B) (Figure 1B). Moreover, and there was a significant difference in the tumor weight between the groups C, D, and B (^****^p < 0.001) (Figure 1C). Immunohistochemistry showed that the expression of Ki67 was lower in groups C and D than that in group B (Figure 1D) and confirmed by the corresponding statistical results (Figure 1E). These results indicate that the growth of osteosarcoma xenograft tumors was inhibited by CDDP and DOX treatment.

### The diversity of species in the gut microbiological environment

**Figure 2:**
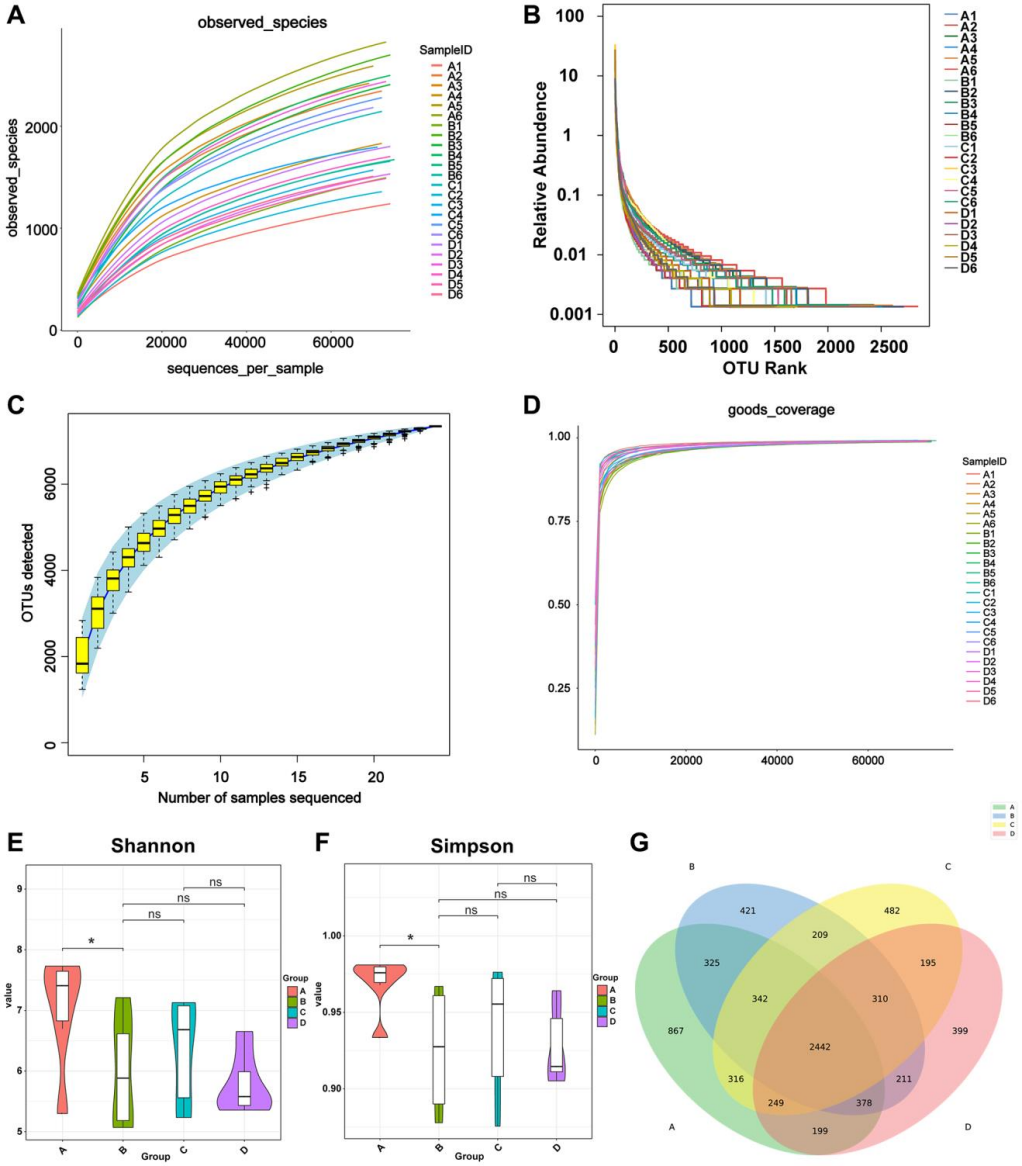
**Calculation of alpha diversity index reflects sequencing depth.** (**A**) The OTUs indicating the numbers of observed bacterial species. The X-axis represents the random sampling depth, and the Y-axis represents the exponential value. The curve slowly plateaus, indicating that there is enough sequencing data to represent information on the majority of microbial species in the sample. (**B**) Species richness and evenness. The abscissa represents the number of sequences contained in OTUs. For example, the 1000^th^ most abundant OTU is represented by “1000”. The ordinate represents the relative abundance of the OTU, where “0.01” stands for 0.01%. (**C**) Species accumulation curve. The number of samples is represented on the X-axis, and the detected OTU number of samples is represented on the Y-axis. The flattening of the curve indicates that the sample size is adequate. (**D**) Good’s coverage analysis. Each sample is represented by one curve. The abscissa represents the random sampling depth, and the ordinate represents the exponential value. The curves flatten early on, and the value of the Y-axis changes barely, indicating the rationality of the amount of sequencing data. (**E**, **F**) Violin diagram of Shannon and Simpson index. Different colors represent different groups, and the ordinate is the index value. (**G**) Venn diagram of the number of OTUs in A, B, C, and D groups. Abbreviation: OTUs: operational taxonomic units.

Bacterial DNA was isolated from fecal samples and 16S rRNA gene sequencing was carried out. First, the quality of the sequencing data was evaluated. The curves of the OTUs, indicating the number of observed species, gradually approached a plateau (Figure 2A), indicating the rationality of the sequencing data. Species evenness and richness between groups are shown in the rank abundance curve (Figure 2B). The species accumulation curve indicates that there was sufficient sampling (Figure 2C). Second, alpha diversity metrics, which included Chao1, PD whole tree, good’s coverage, and Shannon and Simpson indices, were employed to evaluate the community diversity in each sample. Chao1 and phylogenetic diversity (PD) whole tree indices reflected community richness and the relationships among the species within the community, respectively; however, non-significant differences were observed between the four groups (non-tumor bearing, tumor-bearing, CDDP, and DOX). Good’s coverage was used to calculate the probability of a random sequence detected in the sample; thereby, determining the completeness of sequencing. The Good’s coverage index value was close to 1, proving that approximately the entirety of microbial communities was identified in the samples (Figure 2D). The Shannon and Simpson indices represent diversity. The Shannon and Simpson indices of group B were lower than those of group A, while differences between the other groups were not significant (^*^p < 0.05, ns *p* > 0.05; Figure 2E, 2F). This indicated that osteosarcoma growth reduced the species richness and diversity of the host gut microbiota. The Venn diagram (Figure 2G) identified 2442 common OTUs in the four groups of mice, and each group had its own unique OTUs as follows: 867 OTUs were unique to group A, 421 were unique to group B, 482 were unique to group C, and 399 were unique to group D. Mice in the control group had more unique OTUs than those of mice in the osteosarcoma mouse model, and mice treated with CDDP in the osteosarcoma mouse model had more unique OTUs than those of mice treated with saline, indicating that the growth of osteosarcoma reduced the diversity of gut microbiota, which may be partially restored after the CDDP treatment.

### Gut microbial dysbiosis in mice with osteosarcoma

**Figure 3:**
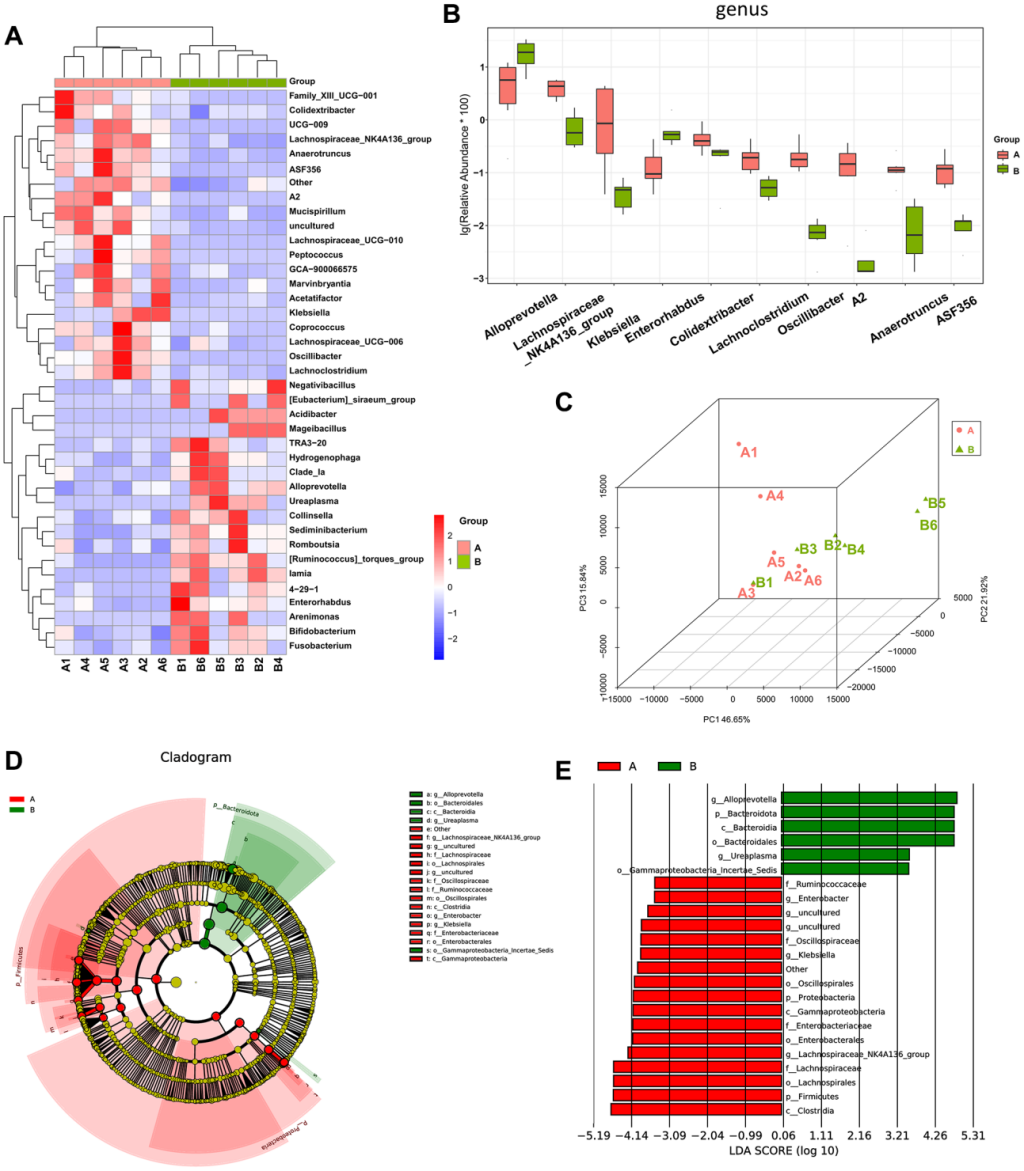
**The difference in diversity of the gut microbiota between mice in the osteosarcoma model group and mice in the control group.** (**A**) Heatmap of genus-level abundance in each sample. The cluster tree on the left represents species clusters and the upper cluster tree represents sample clustering. Red indicates higher relative abundances of the genus, and blue indicates lower relative abundances of the genus. (**B**) Differential genus abundance Top10 boxplot. Different colors represent different groups of samples, and the ordinate represents the log-transformed value of the relative abundance of genera. (**C**) PCoA 3D diagram. Each sample is represented by one point in the Figure, and samples from the same group are of the same color. The closer the samples in the same group are, with a clear distance from other groups, indicates that the grouping effect is good. (**D**) Differential species annotation cladogram from LEfSe analysis. Nodes with different colors, respectively, indicate significantly different species with relatively high abundance in the different groups. Yellow nodes indicate species that were not significantly different between the two groups. The node diameter is proportional to the relative abundance. (**E**) Differential species score chart. Groups are represented by different colors. Genera in group A with a relatively high abundance are displayed in red bars, and green represents group B. Abbreviations: PCoA: principal coordinate analysis; LEfSe: linear discriminant analysis coupled with effect size measurements.

We compared the control group (group A) and the osteosarcoma model group (group B) to reveal alterations in the gut microbiota of mice before and after osteosarcoma growth. Compared to those of group A (control group), the abundance of *Colidextribacter*, *Lachnospiraceae_NK4A136_group*, *Lachnospiraceae_ UCG-010*, *Lachnospiraceae_UCG-006,* and *Lachnoclostridium* was decreased, and the abundance of *Alloprevotella* and *Enorhabdus* was increased in group B (osteosarcoma model group). These results are demonstrated by a heatmap of the differential genera (Figure 3A) and a boxplot of the top 10 differential microbiota at the genus level (Figure 3B). The degree of variation between the different samples was exhibited in the microbial community PCoA (Figure 3C). Samples within a group exhibited a good degree of aggregation, and separation between the two groups of samples was evident. Linear discriminant analysis coupled with effect size measurements (LEfSe) analysis confirmed that *Alloprevotella* had a high degree of enrichment in mice in the osteosarcoma model group, and *Lachnospiraceae* had a high degree of enrichment in mice in the control group (Figure 3D, 3E).

### Effects of CDDP and DOX treatment in the gut microbiota of mice with osteosarcoma

**Figure 4:**
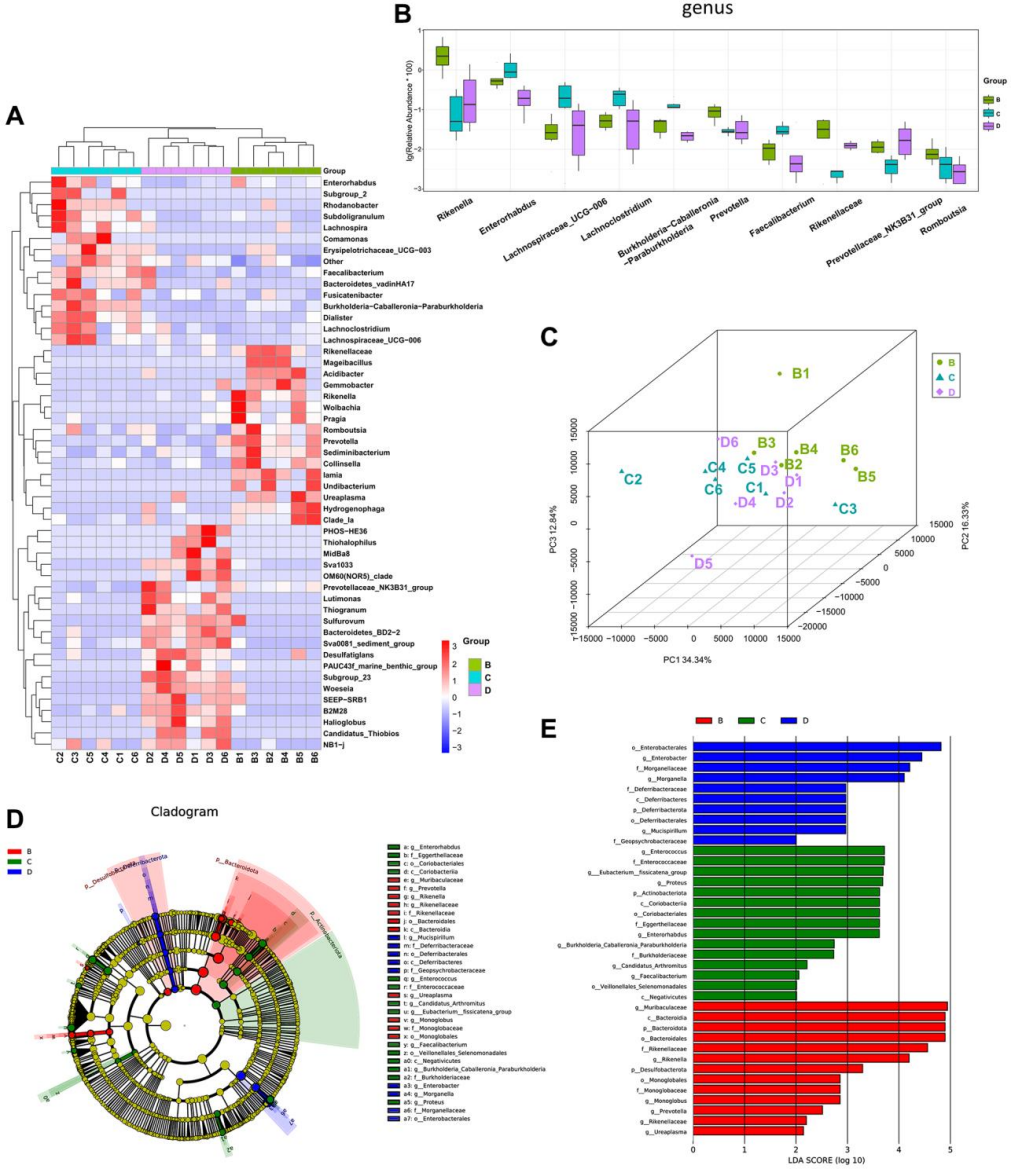
**The different diversity of gut microbiota among CDDP-, DOX- and normal saline-treated mice.** (**A**) Heatmap of genus-level abundance in each sample. (**B**) Differential genus abundance Top10 boxplot. (**C**) PCoA between groups. (**D**) Differential species annotation cladogram from LEfSe analysis. (**E**) Differential species score chart.

CDDP and DOX are commonly clinically used as chemotherapy treatments for osteosarcoma. After five intraperitoneal injections, the effects of CDDP (group C) and DOX (group D) on the gut microbiota of the mice with osteosarcoma were examined. At the genus level, mice in groups C and D exhibited lower abundances of *Rikenella* and *Prevotella* than those of mice in the osteosarcoma model group (group B), *Enterorhabdus*, *Lachnospiraceae_UCG-006*, *Lachnoclostridium*, and *Faecalibacterium* were more abundant in group C (Figure 4A, 4B). PCoA analysis revealed that the gut microbiota in mice in groups C and D differed from that in group B, while the difference of gut microbiota between groups C and D was not significant (Figure 4C). Linear discriminant analysis effect size (LEfSe) analysis confirmed that *Enterorhabdus* and *Faecalibacterium* were highly enriched in group C, and *Rikenella* and *Prevotella* were highly enriched in group A (Figure 4D, 4E).

**Figure 5:**
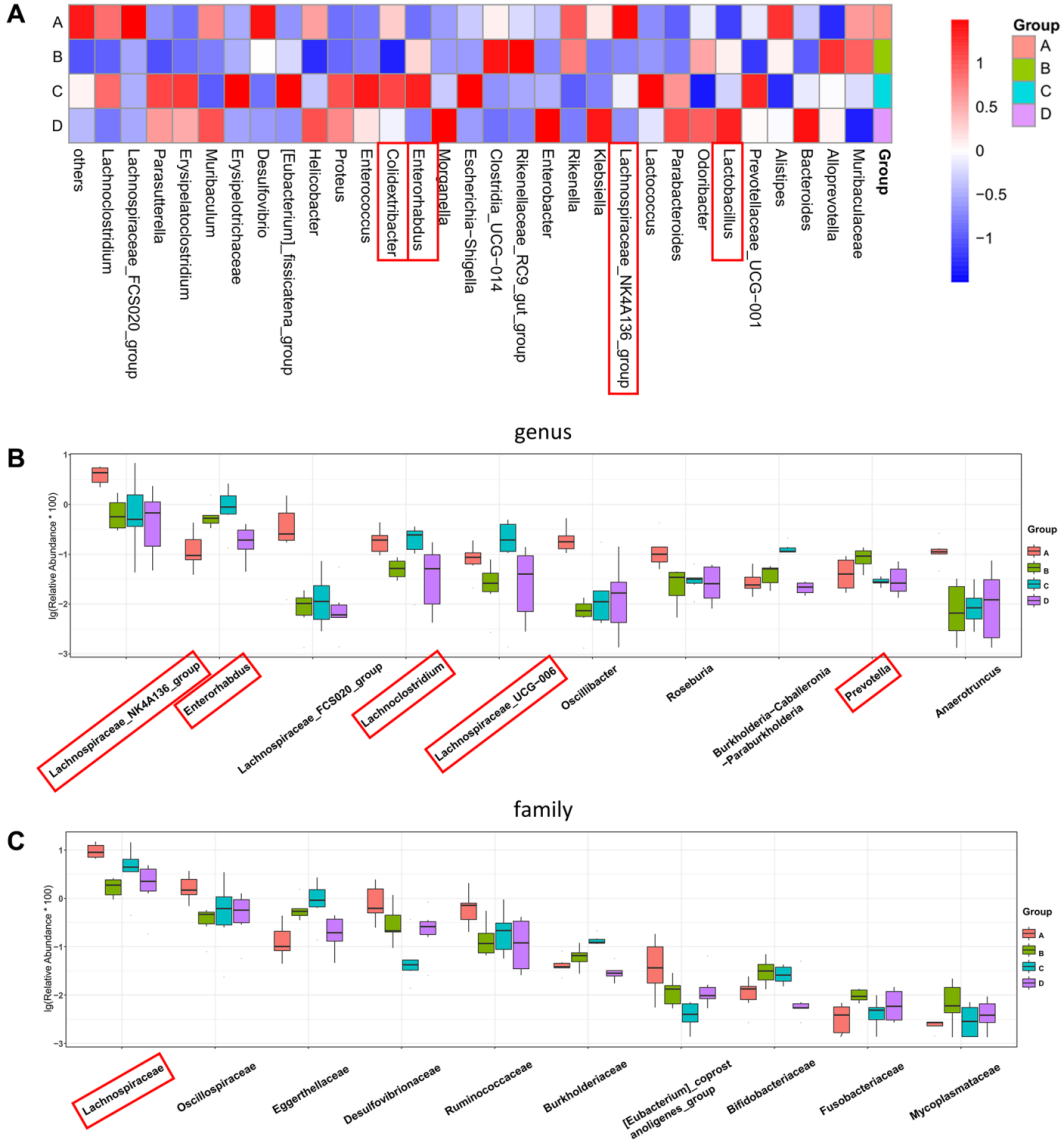
**Four-group comprehensive analysis of changing trends in gut microbiota.** (**A**) Heatmap of genus-level abundance in each group. (**B**) Differential genus abundance Top10 boxplot. (**C**) Differential family abundance Top10 boxplot. Abbreviations: PCoA: principal coordinate analysis; LEfSe: linear discriminant analysis coupled with effect size measurements.

To further investigate the effects of CDDP and DOX treatments on the changes in the composition of the gut microbiota in mice, we included the mice in the control group A for comparison. We observed that the increase in the abundances of *Prevotella* and *Enterorhabdus* in the osteosarcoma mouse model (group B) were reverted to their normal abundances following DOX treatment (group D). The increase in the abundance of *Prevotella* and the decrease in the abundances of *Lachnospiraceae_UCG-006, Lachnoclostridium,* and *Colidextribacter* in group B were also normalized following treatment with CDDP (group C), as indicated by the heatmap for the top 30 differential genera (Figure 5A) and the boxplot for the top 10 differential microbiota at the genus level for the four groups (Figure 5B). In addition, the abundance of *Lactobacillus* significantly increased in the DOX treatment group (group D). At the family level (Figure 5C), *Lachnospiraceae* had the highest abundance.

### Species correlation and phylogenetic analysis

**Figure 6:**
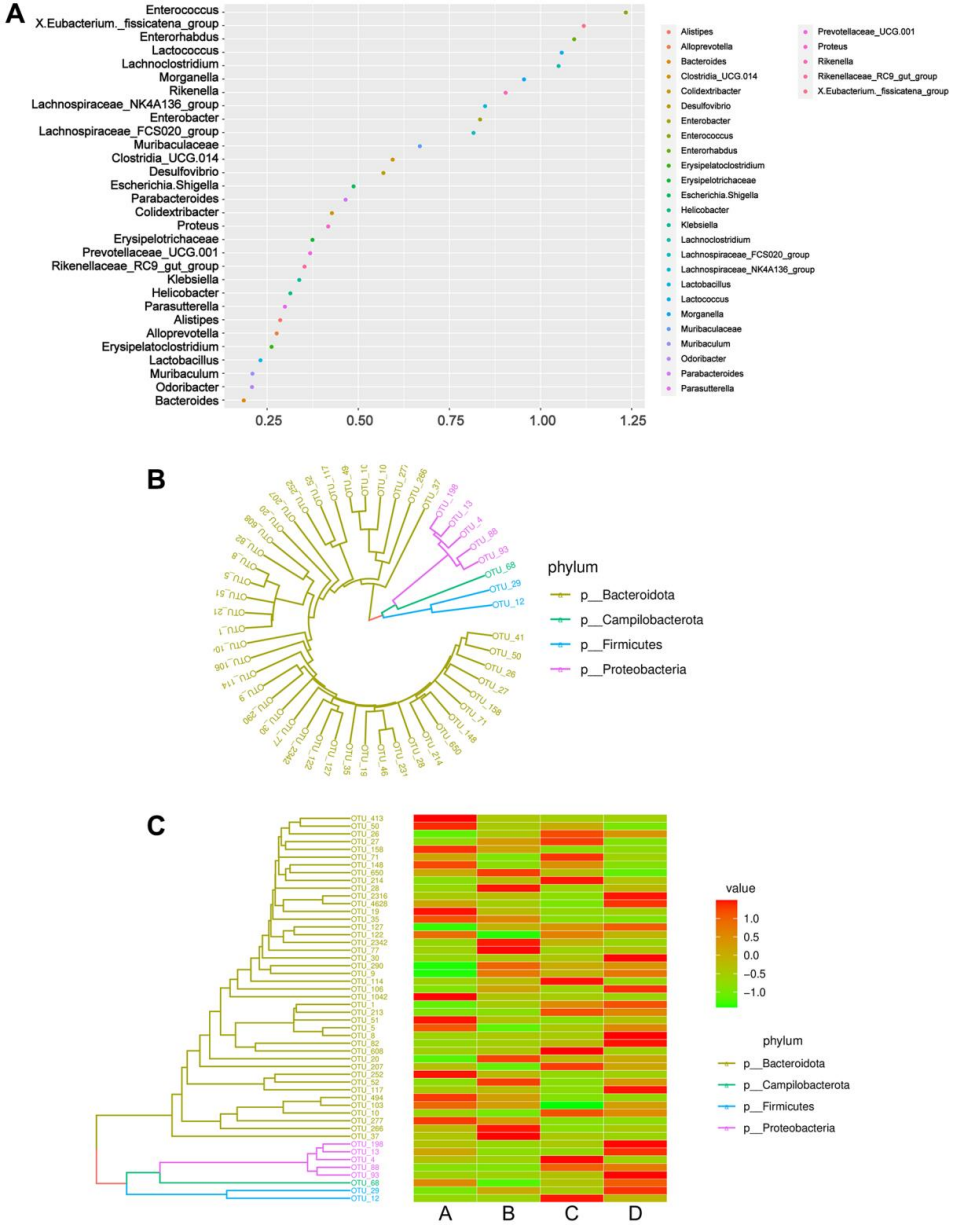
**Phylogeny analysis and classification of microbiome community samples.** (**A**) Genera importance point plot. The X-axis represents the measure of importance, and the Y-axis is the genera names ranked by importance. Normalized importance values are used by default in the plot. (**B**) Phylogenetic tree diagram. Different colors correspond to different phyla. (**C**) Combination diagram of species abundance and phylogenetic tree. The left is the phylogenetic tree diagram; the right is the abundance map, corresponding to the abundance of the OTU on the left in each group. Abbreviation: OTUs: operational taxonomic units.

Phylogeny studies the formation or evolutionary history of species and the evolutionary relationships among species. In molecular evolutionary research, phylogenetic inference can reveal the sequence of biological evolutionary processes, understand the biological evolutionary history and mechanisms, and build an evolutionary tree based on the base differences among sequences at a certain taxonomic level. A random forest diagram was constructed to predict species richness (Figure 6A). As a machine learning algorithm, random forest is capable of efficiently and accurately classifying microbial community samples and can identify key components (OTUs or species) that can distinguish the differences among groups. Using the OTU abundance counts, we selected the TOP50 OTUs with the highest abundance, constructed an evolutionary tree (Figure 6B), and displayed the abundance of OTUs in different samples using a heatmap (Figure 6C). Compared to those of the CDDP and DOX groups (groups C and D, respectively), lower abundances of *Campilobacterota*, *Firmicutes,* and *Proteobacteria* were observed in the osteosarcoma mouse model group (group B).

### Treatment with CDDP or DOX in mice in the osteosarcoma mouse model induced changes in the inferred gut microbiota function

**Figure 7:**
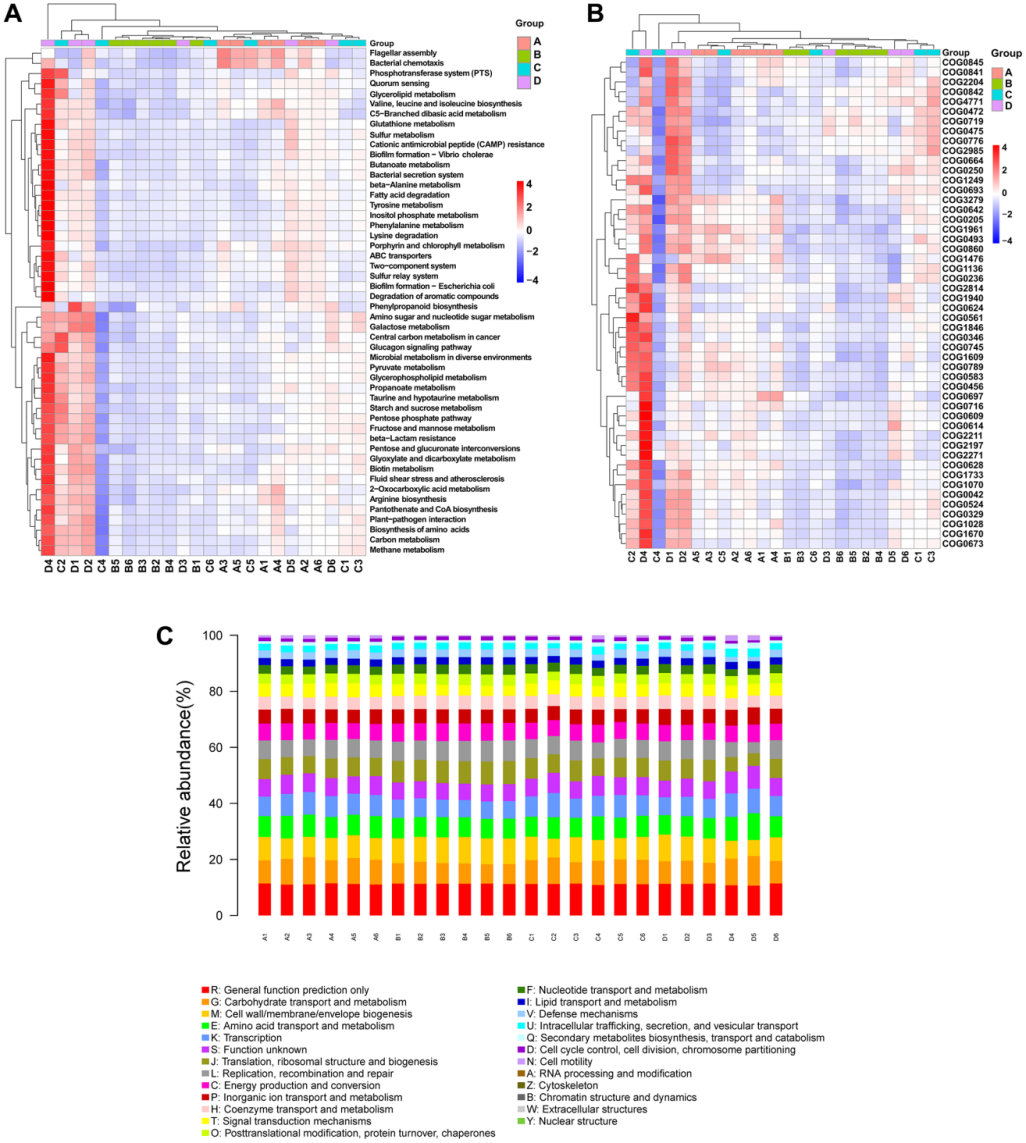
**PICRUSt2 functional predictive analysis.** (**A**) KEGG difference clustering heatmap. Kruskal–Wallis analysis was performed to predict pathways. The clustering of different enrichment entries is exhibited in the left cluster tree, and the upper clustering branch represents different samples. (**B**) COG difference clustering heatmap. (**C**) COG bar plot. The sample names are on the X-axis, and the relative abundance of the predicted functions is on the Y-axis. Abbreviations: PICRUSt2: phylogenetic investigation of communities by reconstruction of unobserved states; KEGG: Kyoto Encyclopedia of Genes and Genomes; COG: Clusters of Orthologous Groups of proteins.

PICRUSt2 was used to predict differences in gut microbiota function based on differences in the gut microbiota composition. Briefly, 50 pathways were identified from the Kyoto Encyclopedia of Genes and Genomes (KEGG) database, most of which are related to metabolism (Figure 7A). The analysis indicated that the osteosarcoma model group (group B) had lower heatmap scores for the metabolism of carbon, nucleotide sugar and amino sugar, starch and sucrose, butanoate, propanoate, glutathione, and amino acids (phenylalanine and tyrosine), the biosynthesis of amino acids (arginine, valine, leucine, and isoleucine), and the degradation of fatty acids in comparison to those of the control group (group A). These pathways were all elevated in the CDDP- and DOX-treated groups compared to those in the osteosarcoma model group. Based on the NCBI Clusters of Orthologous Groups of proteins (COG) database, we carried out a difference analysis on the predicted COG results using the top 30 entries in the difference results and displayed them in a heatmap (Figure 7B). The prediction of potential functions of the microbiome is also presented in a bar plot (Figure 7C), identifying some COG entries with high relative abundances related to microbial changes, such as carbohydrate transport and metabolism, amino acid transport and metabolism, and transcription.

## DISCUSSION

Gut microbiota plays an essential role in health and disease. Gut microbiota and their associated metabolites are closely related to carcinogenesis, as they can induce inflammation and immune dysregulation and therefore, can interfere with the pharmacodynamics of anticancer agents [27]. We demonstrated that the presence of osteosarcoma and treatment with CDDP or DOX altered the composition of the gut microbiota in mice.

In this study, the growth of osteosarcoma xenografts led to dysbiosis of the gut microbiota, reducing the species richness and diversity. The microbiota dysbiosis observed in mice with osteosarcoma compared to that in control mice included an increase in the abundances of *Alloprevotella* and *Enterorhabdus* and a decrease in the abundances of *Colidextribacter* and the four genera of *Lachnospiraceae*. Despite reports on its potential beneficial attributes, *Alloprevotella* is a member of the *Prevotellaceae* family that has been previously associated with colorectal cancer and autoimmune diseases [28, 29]. The abundance of *Enterorhabdus*, a harmful bacterium, was reported to be elevated in mice in a non-alcoholic steatohepatitis model. *Colidextribacter* produces inosine [30], which is involved in energy metabolism as a physiological energy source and possesses a wide range of anti-inflammatory and immunomodulatory properties, including promoting anti-inflammatory cytokine production and inhibiting the production of chemokines and pro-inflammatory factors [31]. Many genera of *Lachnospiraceae* can produce butyrate [32], a short-chain fatty acid that is a major source of energy for intestinal epithelial cells, which can also inhibit pro-inflammatory cytokine signaling pathways [33]. Additionally, butyrate can enhance tight junctions and increase mucin secretion, thereby enhancing intestinal barrier function [33]. Dysbiosis or alteration of specific microbes may be caused by cancer and may lead to altered homeostasis or epigenetic effects that contribute to tumor progression and cellular transformation [34, 35]. The gut microbiota is also associated with cancer cachexia through pathways involved in systemic inflammation and gut barrier dysfunction [36]. Therefore, we believe that dysbiosis of the gut microbiota in mice, an increase in number of harmful bacteria and a decrease in number of beneficial bacteria, may promote osteosarcoma progression.

We found that treatment with CDDP or DOX altered the composition of gut microbiota in mice with osteosarcoma. Treatment with CDDP or DOX reduced the abundance of *Rikenella*, and CDDP increased the abundance of *Faecalibacterium*. The chemotherapy drug cyclophosphamide also reduces the abundance of *Rikenella*, a beneficial bacterium that can produce short-chain fatty acids [37], which may be a side effect of chemotherapy drugs. Patients with a high abundance of *Faecalibacterium* experience higher systemic and antitumor immune responses mediated through increased antigen presentation and exhibit improved effector T cell function in the tumor microenvironment and its periphery than those of patients with a low abundance of *Faecalibacterium* [38]. Iida et al. found that treatment of subcutaneous transplanted tumors with the platinum compounds oxaliplatin and cisplatin in antibiotic-treated or germ-free mice was significantly less effective than that in conventional mice [39]. Additionally, we noted that levels of some beneficial bacteria decreased during the growth of osteosarcoma tumors and were increased after chemotherapy, suggesting that the chemotherapy treatment may have corrected the cancer-induced changes in levels of beneficial bacteria, including *Lachnospiraceae_UCG-006*, *Lachnoclostridium,* and *Colidextribacter*. These results indicate that changes in the gut microbiota after chemotherapy may contribute to the antitumor effects in the body.

In addition, we performed 16S-based KEGG and COG function predictions. The gut microbiota produces or alters various chemicals and can trigger various host responses that affect physiological functions, including immunity, neurobiology, and metabolism [40, 41]. The KEGG pathway prediction results indicated that metabolism-related pathways accounted for the largest proportion of potentially affected pathways, including butanoate metabolism. Butyrate boosts antitumor CD8^+^ T cell responses through ID2-dependent IL-12 signaling [42]. The tumor-suppressive effect is increased by combination treatment with butyrate and chemotherapy or immunotherapy, indicating that gut microbiota metabolite supplementation may have implications for cancer treatment responses [42]. Previous studies have demonstrated that sodium butyrate has anti-proliferative, pro-apoptotic, pro-differentiation, and immunomodulatory effects on osteosarcoma cells [43, 44]. In this study, butanoate metabolism was high in mice in the control group, decreased in mice in the osteosarcoma model group, and increased in osteosarcoma mice after CDDP or DOX treatment. The abundance of *Lachnospiraceae*, a butyrate-producing bacterium, showed a similar pattern. Therefore, we suggest that alterations in butyrate metabolism and *Lachnospiriaceae* levels may play important roles in the progression and chemotherapy of osteosarcoma, and further research is warranted. The COG prediction results also suggested that there is a significant relationship between changes in the gut microbiota and metabolism-related functions, including carbohydrate transport and metabolism and amino acid transport and metabolism. However, compared to the analysis using metabolomics, PICRUSt2 analysis still has certain drawbacks, which is a limitation of this study.

Significant reductions in butyrate-producing bacteria have been observed in colorectal cancer patients [45]. Furthermore, supplementation with *Clostridium butyricum*, a butyrate-producing bacteria commonly used in clinical settings, inhibits intestinal tumor development in mice by modulating Wnt/β-catenin signaling and gut microbiota [45]. A recent phase I clinical trial in refractory metastatic melanoma showed that fecal microbiota transplantation (FMT) from responding patients can restore the effects of anti-PD-1 immunotherapy in non-responding patients [46]. This study suggests that bacteria, such as *Lachnospiraceae* and *Colidextribacter* may play an important role in the growth and treatment of osteosarcoma. Whether FMT or supplementation with certain bacteria in the gut may be a potential novel addition to adjuvant therapy for osteosarcoma remains to be further explored.

## CONCLUSIONS

In this study, we found that osteosarcoma growth altered the composition of gut microbiota in mice, and the levels of several bacterial populations were further altered after treatment with CDDP or DOX. KEGG and COG analyses showed that these changes were closely related to a variety of physiological functions in mice. This study helps establish the link between the gut microbiota and osteosarcoma growth and chemotherapy; also, provides the basis for further studies on the involvement of the gut microbiota in osteosarcoma progression and chemotherapy that may lead to the development of potential new therapeutic strategies.
